# Methods for Differentiating Prion Types in Food-Producing Animals

**DOI:** 10.3390/biology4040785

**Published:** 2015-11-13

**Authors:** Kevin C. Gough, Helen C. Rees, Sarah E. Ives, Ben C. Maddison

**Affiliations:** 1 School of Veterinary Medicine and Science, The University of Nottingham, Sutton Bonington Campus, College Road, Sutton Bonington, Leicestershire LE12 5RD, UK; E-Mail: svysei@exmail.nottingham.ac.uk; 2 ADAS UK, School of Veterinary Medicine and Science, The University of Nottingham, Sutton Bonington Campus, College Road, Sutton Bonington, Leicestershire LE12 5RD, UK; E-Mails: Helen.Rees@adas.co.uk (H.C.R.); Ben.Maddison@adas.co.uk (B.C.M.)

**Keywords:** prion, transmissible spongiform encephalopathy, scrapie, BSE, CWD, strain typing

## Abstract

Prions are an enigma amongst infectious disease agents as they lack a genome yet confer specific pathologies thought to be dictated mainly, if not solely, by the conformation of the disease form of the prion protein (PrP^Sc^). Prion diseases affect humans and animals, the latter including the food-producing ruminant species cattle, sheep, goats and deer. Importantly, it has been shown that the disease agent of bovine spongiform encephalopathy (BSE) is zoonotic, causing variant Creutzfeldt Jakob disease (vCJD) in humans. Current diagnostic tests can distinguish different prion types and in food-producing animals these focus on the differentiation of BSE from the non-zoonotic agents. Whilst BSE cases are now rare, atypical forms of both scrapie and BSE have been reported, as well as two types of chronic wasting disease (CWD) in cervids. Typing of animal prion isolates remains an important aspect of prion diagnosis and is now becoming more focused on identifying the range of prion types that are present in food-producing animals and also developing tests that can screen for emerging, novel prion diseases. Here, we review prion typing methodologies in light of current and emerging prion types in food-producing animals.

## 1. Introduction

### 1.1. Transmissible Spongiform Encephalopathies, and the Concept of Prions as an Infectious Agent

The transmissible spongiform encephalopathies (TSEs), commonly referred to as prion diseases, are a group of infectious, fatal neurodegenerative disorders that affect a number of mammalian species, including man. The prion hypothesis proposes that the infectious agent is largely comprised of the PrP^Sc^ conformer of a benign host encoded membrane glycoprotein (PrP^C^) in the absence of any coding nucleic acid [1]. The conformational rearrangements (protein misfolding) that accompany conversion of PrP^C^ to PrP^Sc^ result in increased insolubility in detergents [2] and protease resistance [3]. PrP^C^ has a wide tissue distribution and is present as di-, mono- and un-glycosylated forms. It is thought that PrP^Sc^ is responsible for the conversion of additional molecules of PrP^C^ into PrP^Sc^ and that this is the central molecular event that occurs during TSE disease. This conversion event is largely restricted to those cells of the lymphoreticular tissues (LRS) and central nervous system (CNS), and the end product has a propensity to aggregate and form amyloid. It is unclear as to the effect of the loss of function of PrP^C^ that occurs after conversion to PrP^Sc^ as the physiological function of PrP^C^ remains a controversial matter. However, it is thought that either this PrP^Sc^ end product, or a conformer of this protein formed along the folding pathway is toxic to cells of the CNS and is responsible for the characteristic brain pathology including the spongiosis, astrocytosis and neuronal loss that are commonly associated with these diseases.

### 1.2. Examples of Prion Diseases

Prion diseases occur in humans, for example Creutzfeldt-Jakob disease (CJD) and kuru. They also occur in food-producing animals (Table 1), namely the ruminant species sheep, goats, cattle and deer. Sheep and goats are susceptible to the prototypic prion disease, classical scrapie, as well as atypical/Nor98 scrapie, cattle and goats are susceptible to classical bovine spongiform encephalopathy (BSE) and cattle also to atypical BSE types L- and H-type, and cervids are affected by chronic wasting disease (CWD). Some prion diseases are inherited whilst others can be spontaneous or be acquired from an extraneous source. Prion diseases of ruminants are usually the latter, for example classical BSE infection of cattle arose from the consumption of BSE contaminated feed, and in sheep and goats classical scrapie may be transmitted via direct contact with infected animals or their environs. However, it has been suggested that atypical BSE and atypical/Nor98 scrapie diseases may occur spontaneously [4,5,6,7]. Prion diseases became the focus of intense research following the UK BSE epidemic in cattle in the late 1980s and 1990s, and then the identification of this as the causal agent of variant CJD (vCJD) in humans. The zoonotic potential of this class of disease causing agents along with the emergence of several newly recognised TSEs in recent years, notably atypical types of both scrapie and BSE, illustrate the requirement for continued research and surveillance into TSEs of food-producing animals. This includes the development of methods for their detection and differentiation. 

**Table 1:** Prion types affecting farmed ruminants.

Prion types *	Host Species
Classical BSE (bovine spongiform encephalopathy)	Cattle, Goats ^#^
Atypical BSE: H-BSE, L-BSE/bovine amyloidotic spongiform encephalopathy (BASE)	Cattle
Classical Scrapie ^^^	Sheep, Goats
CH1641 Scrapie ^+^	Sheep
Atypical/Nor98 Scrapie	Sheep, Goats
CWD (chronic wasting disease)	White-tailed deer, Elk, Mule Deer, Moose

* Types as defined through analysis of tissue taken from food production animals. ^#^ BSE in goats is very rare, to date only 2 cases of BSE have been recorded in this species. ^^^ Further characterisation of classical scrapie isolates in inbred strains of mice have demonstrated that the classical scrapie category can comprise over 15 distinct scrapie strains. ^+^ CH1641 scrapie is a rare form of scrapie found in sheep that shares similar biochemical features to that of BSE.

### 1.3. TSE Diagnosis

TSEs often present as a spectrum of different clinical signs in the infected animal. In scrapie-affected sheep and goats, infection may show as pruritus, weight loss, wool pulling, the swaying of hips and hind limbs, and animals can develop a body tremor and have an increased sensitivity to noise and movement. As disease progresses animals can develop an inability to stand, and exhibit other behavioural changes. In BSE affected cattle clinical observations may be subtle or obvious and can include changes in temperament such as nervousness or aggression, animals can also be seen to develop an abnormal posture, demonstrate a lack of coordination and have difficulty in rising. There is decreased milk production, and animals often show weight loss. With CWD, clinical signs may include emaciation, an excessive salivation, ataxia, the drooping of head and ears, weakness and behavioural changes. 

**Figure 1:**
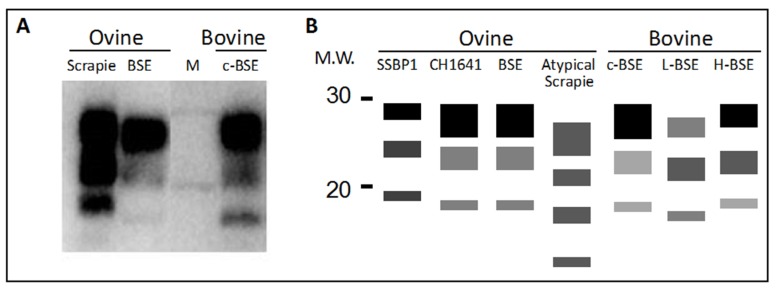
(**A**) Example western blot using a core antibody to detect prion protein (PrP^Sc^) after proteinase K digestion showing the typical di- mono- and unglycosylated protein banding pattern. Ovine scrapie, ovine bovine spongiform encephalopathy (BSE) and bovine classical BSE (c-BSE) are shown, M, molecular mass markers 20 and 30kDa. Evident are the lower molecular weight of the unglycosylated protein band in ovine or bovine BSE compared to scrapie. Also, the di-glycan band is more dominant in BSE compared to the scrapie sample. A schematic diagram (**B**) to illustrate examples of the different electrophoretic glycoform profiles of the PrP^Sc^ from different prion types following Proteinase K digestion and western blotting using an antibody to the core region of the prion protein. Ovine SSBP1 is readily differentiated from both CH1641 scrapie and BSE, by glycoform ratio and molecular weight of the unglycosylated PrP^Sc^ species. Atypical forms of scrapie are less protease resistant than classical scrapie and typically show lower molecular weight species. The unglycosylated PrP^Sc^ band in classical bovine BSE is intermediate in size between that of L-type and H-type atypical forms of bovine BSE. M.W. Molecular mass markers in kDa. It should be noted that for ovine CH1641and bovine H-BSE, evidence for two different conformer populations are present within these profiles. These are revealed upon independently probing blots with the core antibody SAF84 and a second antibody that binds more towards the N terminus (for example L42), and evidenced by differences within the glycoprofiles and banding patterns between these blot [8,9], also see sections 4.1 and 4.2. This is a unique trait of these two prion types.

For all ruminant prion diseases, clinical signs are not specific enough for diagnosis and often these signs may be subtle or go unnoticed. The most common methods for diagnosing prion diseases rely on differentiating between PrP^C^ and PrP^Sc^ in post mortem samples by their distinct resistances to proteinase K (PK): under defined conditions, PrP^C^ is completely degraded and PrP^Sc^ is cleaved at the N-terminus leaving a protease resistant core of 27 to 30 kDa (Figure 1). PrP^Sc^ is so far the only validated marker for TSE infection. Post-mortem tissue is usually examined for the presence of PrP^Sc^ by either western blot or ELISA methods after PK treatment, or by immunohistochemical (IHC) staining techniques that define foci of PrP^Sc^ aggregation in the absence of protease treatment. 

### 1.4. The Concept of Prion Strains and Species Barrier

Prion strains are defined TSE isolates that when transmitted to a specific host species (usually an inbred strain of mouse) exhibit phenotypic disease traits that are distinct from one another. These traits include differences in disease incubation time, the lesion profile and IHC staining pattern within the CNS, and the biochemical characteristics of the PrP^Sc^ [10]. A characteristic of prion strains is that these traits are stable over serial transmission within that same host species. The basis of TSE strains may be explained by the notion that PrP^Sc^ can adopt different conformations within the secondary, tertiary or quaternary structure of the protein. Experimental evidence illustrating that TSE strains are likely to be made up of different conformational forms of PrP^Sc^ has been repeatedly demonstrated by the different strain-to-strain properties of the PrP^Sc^ protein with regard to protease digestion with PK [11] and treatment with different chaotropic agents [12]. Within TSE surveillance the term strain typing is often used to refer to the description of the biochemical properties of PrP^Sc^ from a particular isolate, however the true identification of a TSE strain can only be confirmed by determining a stable disease phenotype in specific lines of inbred rodent strains. 

The ability of a TSE to infect a distinct host species is governed by a phenomenon commonly referred to as the species barrier [13]. When prion-containing material is inoculated experimentally from one species into another, for example an isolate of sheep scrapie being inoculated into an inbred mouse line, the incubation time for the development of TSE will often be long and there will be an incomplete attack rate (only a proportion of the mice inoculated will develop disease). This inefficient transmission is the species barrier. However, if brain material from these infected animals from this first passage is then further inoculated into more animals of the same genetic background, the attack rate will increase and the incubation time to disease will reduce. On subsequent passage this incubation time will stabilise to produce consistent and reproducible incubation times, hit rates and other phenotypic properties. The apparent “species barrier” between the inoculum from the donor species and the recipient species will have been overcome. Species barriers can also be overcome where homologous PrP^C^ is expressed in the host and the infecting inoculum, as is the case when using transgenic rodents for bioassay [14]. The nature of the TSE agent also contributes to the species barrier. An example is BSE, this TSE of cattle is experimentally transmissible to a wide variety of species with diverse PrP^C^ primary structures [15] and in natural transmissions it has caused prion disease in a number of species including humans, domestic cats (FSE) and exotic ungulates [16], presumably as a result of ingestion of BSE-contaminated food. This is in contrast to scrapie, where a similar variety of species would have been exposed to the scrapie agent, yet this disease has only been seen in natural infections of sheep and goats.

### 1.5. The Emergence of “New” Prion Strains

**Figure 2:**
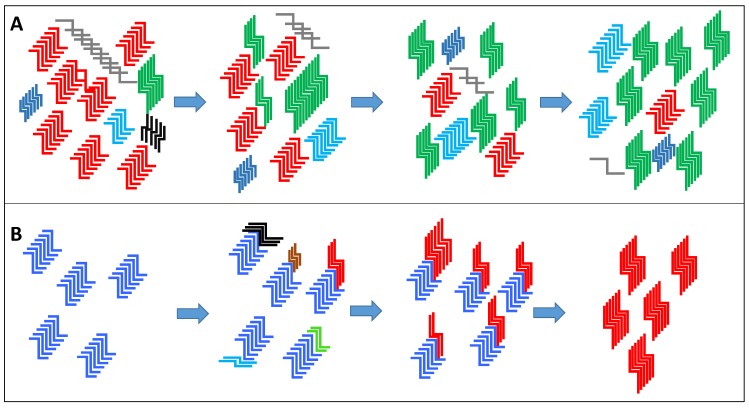
(**A**) The premise of the “cloud” hypothesis is that a prion type is comprised of a heterogeneous mix of PrP^Sc^ variants made up of a dominant conformer (the example given is depicted in red) and minor populations of PrP^Sc^ variants (other colours). A change in the replication environment or an environmental pressure can then select for a minor variant within the “cloud” to become the dominant type over time (the example of a new dominant PrP^Sc^ conformer is depicted in green). (**B**) The deformed template theory proposes that new PrP^Sc^ structural variants are created directly from the original dominant conformer (blue) due to a new environment for replication. Variants are generated from a number of trial and error seeding events and one that is particularly suited to the new replication environment becomes the new dominant conformer (red) replacing the original variant (blue) (figure adapted from Makarava and Baskakov [18]).

Whilst it is easy to regard prion strains as a fixed entity, several lines of experimental evidence suggest that this is an oversimplification. ‘New’ strains with different properties to that in the inoculum have been seen to emerge after transmission to different species, even when the inoculum had been cloned by limiting dilution [17]. The emerging view is that a prion strain may exist much like a quasispecies for viral and bacterial pathogens. The cloud hypothesis [13] proposes that a wide range of PrP^Sc^ conformers are possible within a given strain, but that within each prion ‘strain’ only a subset of those conformers are able to interact with the host PrP^C^ that is presented within any given infection. Thus there will be a range of PrP^Sc^ infectious conformers that are able to become the dominant species upon transmission within a new host. For an efficient infection to proceed the PrP^C^ from the host has to be capable of forming a conformation that is compatible with the infecting PrP^Sc^ strain. Thus the selection of the new dominant species is dependent on the appropriate conditions being present for its selection. The selection of a minor conformer within a newly infected host and its preferential replication over the previous dominant ‘strain’ would go some way to explaining how prion strains can apparently mutate (Figure 2). 

A complementary theory has also been proposed called “the deformed template theory”, suggesting that the replication environment not only influences the evolution of existing PrP^Sc^ conformations to become the dominant strain, but actually plays an active and vital role to produce novel *de novo* PrP^Sc^ conformations (Figure 2). It is likely that the cloud hypothesis and deformed template theory are both relevant, the main differences between the two theories are that in the deformed template theory the environment has an active part in the creation of novel PrP^Sc^ conformations and that the original or “parent” prions act as a template for the creation of such *de novo* prion strains [18]. 

Studies have suggested that novel synthetic prions can be created *de novo*; a transmissible prion strain being generated after inoculation of rodents with recombinant-PrP amyloid fibrils. This could give further support to the deformed template theory with PrP^Sc^ possibly being formed from the original (non-PrP^Sc^) fibrils [19,20,21]. Using an *in vitro* prion replication method (protein misfolding cyclic amplification; PMCA) to amplify prions from a scrapie-infected brain sample, another study showed that when RNA was removed from and then returned to the replication environment, a novel prion strain developed which was not present in the original brain sample, further supporting the deformed template theory [22].

### 1.6. The Significance of New Strain Emergence

Prion strains can appear to undergo Darwinian selection given the right selective pressures. Ghaemmaghami and co-workers [23] have demonstrated the *in vitro* and *in vivo* selection of novel prions in both cell culture and in mice, using biologically cloned murine adapted prions. In these experiments prions could be seen to adapt to the selection pressures of either brain (infection of mice) or cell culture replication where the action of the anti-prion compound quinacrine could select for resistant prions. In addition, Li and co-workers [24] demonstrated that the rodent scrapie strain 22L changed its dominant conformer and concomitant disease phenotype due to selective pressure exerted by the presence of swainsonine which disrupts N-linked glycosylation. These observations support the notion of a prion quasispecies and prion strain selection; they are also extremely important to our understanding of these diseases and the potential zoonotic risk that these diseases may pose in the future. 

A recent study used transgenic mouse bioassay to examine a mixture of ovine scrapie isolates before and after incubation on soil for 13 months to determine whether the dominant types were consistent during this incubation period within an environmental matrix [25]. The data showed that prolonged ageing of scrapie prions within the soil resulted in changes in the dominant scrapie pathologies and concomitant PrP^Sc^ biological/biochemical properties. This data indicates that environmental reservoirs of ruminant prions may be dynamic and the dominant “strain” may change over time potentially even leading to the emergence of novel prions. Understanding how TSE strains are maintained within populations and how they might have the ability to cause new infections within new hosts or after prolonged incubation in environmental reservoirs given the correct selective pressures is of importance if we are to minimise the potential for these agents to affect human health.

### 1.7. Prion Diseases that Affect Food-Producing Animals

#### 1.7.1. BSE

BSE was first discovered in 1986 in UK cattle [26] and led to the BSE epidemic in the UK. One of the striking traits of this epidemic was the homogeneity of the BSE agent which led to the conclusion that there was only one type of TSE involved. However, the discoveries of BSE cases with features that were different from classical BSE have now been reported and are referred to as atypical BSE [27,28,29,30]. These cases of atypical BSE can be separated into H-BSE so called due to the appearance of an unusually high molecular weight of PK-resistant PrP^Sc^ as compared to classical BSE [27,28,30], and L-BSE so called due to the appearance of PK-resistant PrP^Sc^ with a lower molecular weight than classical BSE together with a lower proportion of the diglycosylated form [29] (Figure 1). L-BSE is also termed bovine amyloidotic spongiform encephalopathy (BASE) as it is characterised by the presence of amyloid plaques not observed in classical BSE. Following widespread surveillance of small ruminants in the European Union, classical BSE has been found in two cases in goats [31,32] but not in sheep [11].

#### 1.7.2. Scrapie

Classical scrapie in sheep and goats is the prototype TSE and is thought to have existed for hundreds of years. An atypical scrapie type in sheep was found in Norway in 1998 and designated Nor98, now commonly known as “atypical scrapie" or atypical/Nor98. This differs from classical scrapie in several ways, including the neuroanatomical distribution of histopathological lesions and the pattern of PrP^Sc^ deposits in the brain [33]. In 2002, an obligatory active surveillance programme for scrapie in the sheep population submitted for abattoir slaughter was implemented and has resulted in an increased diagnosis of classical scrapie across the EU. Additionally, scrapie cases in sheep with the newly discovered atypical/Nor98 characteristics have been widely reported [7,34,35]. 

In 2007, an atypical/Nor98 scrapie case was found in a Swiss goat [36] the findings of the study suggested that cases in goats may have been missed in the past and may continue to be under-represented due to the routine sampling and analysis of caudal brainstem and cerebellum. The latter is a major site of PrP^Sc^ deposition in atypical/Nor98 scrapie of sheep but not of goats. Recently a study has proposed the co-existence of classical and atypical/Nor98 scrapie types due to the finding of an unusual case in Italy in which a sheep showed immunohistochemical and PrP^Sc^ features different from other atypical/Nor98 cases [37].

#### 1.7.3. CWD

CWD was first identified in captive deer in Colorado in 1967 and has now been described in both captive and wild cervid populations across the US, in three Canadian provinces, and in the Republic of Korea. Two strains of CWD have been reported in mule deer, Rocky Mountain Elk, white-tailed deer, and moose [38,39]. 

## 2. Typing of TSEs in Food-Producing Animals

Overall, there are a considerable number of TSE types that infect ruminants (Table 1). Whilst scrapie, BSE and CWD appear to be mostly constrained to ovines, bovines and caprines respectively, the occurrence of natural infections of BSE in multiple mammalian species [32,40,41,42] indicates the potential for these infectious agents to cross species barriers. In addition, there are multiple types of each of these TSEs. The ability to define and diagnose individual types of TSE agent is of importance to understand the epidemiology, transmission and pathology of these diseases in farmed animals. Prion typing also underpins the testing of food products to protect human health by ensuring zoonotic BSE prions are not present and by monitoring for the emergence of any novel TSE types. This review will report the most up to date advances in the prion typing of natural TSE infections of ruminants.

### 2.1. Rodent Bioassay: Wild-Type Mouse Lines

Prion strains are defined by their distinct and reproducible patterns of replication after serial passage in mouse lines. Serial passage experiments in mice of scrapie, BSE and CWD have identified over 20 distinct TSE strains, with at least 15 different scrapie strains [10], and can allow the discrimination of BSE from scrapie. The mouse model is commonly used to demonstrate differences in the incubation time (time from inoculation to the onset of clinical symptoms) and brain pathology. This is because, within a particular TSE strain, the genetic background of the mouse model used leads to different incubation periods. Thus, consistency in incubation time becomes a good marker for a particular TSE strain within a particular genetic background of murine host [43]. In terms of the observed neuropathology, different TSE strains can cause marked differences in the severity and distribution of the pathological changes within the brain tissue, due to the different neuroanatomical targeting of each strain. As a semi-quantitative measure of strain discrimination, the severity of the tissue vacuolation as observed by hematoxylin and eosin staining is generally scored from sections of nine grey matter [44] and three white matter brain areas [43]. This data is usually plotted to form a lesion profile, reporting the severity of lesions being scored against each brain area. The plot that is generated shows characteristics of that particular TSE strain within that mouse line. 

A more detailed analysis of the neuropathology and the cell types that are involved can be carried out by immunostaining sections of brain for PrP^Sc^ within areas of vacuolation. TSE strains demonstrate reproducible differences from each other in the extent of this staining [45]. Accumulation of PrP^Sc^ at different foci within the tissue may be in the form of diffuse deposits within the areas of vacuolation or may appear as amyloid plaques [10]. The basis of the differences in the observed neuropathology is thought to be due to the differences in the ability of each prion strain conformer to replicate within the different cell populations, and this in part may be coincident with subtle differences in the structure of PrP^C^ within these sites. 

Without doubt, mouse bioassays have provided a validated method for prion strain discrimination, including the analysis of a range of isolates from natural ruminant hosts. The biggest drawback of using wild-type mice for the identification of distinct prion strains from natural hosts are the long incubation periods and incomplete attack rates that are often associated with the species barrier between the inoculums and the murine host on primary passage.

### 2.2. Rodent Bioassay: Transgenic Mouse Lines

To date, around 20% of classical scrapie isolates and all atypical/Nor98 scrapie isolates are unable to transmit to the wild type mouse strains that are used for prion typing [46]. Many of the deficiencies seen with inbred mouse lines can be resolved by the availability of transgenic lines of mice that either express a PrP homologous to the natural host from which the TSE inoculum originated or that express much higher levels of rodent PrP. The result of this is an enhancement in the susceptibility to natural field isolates and in general greatly reduced incubation times. Transgenic lines are therefore extremely useful in the analysis of prions from ruminant host and are contributing to a better estimate of the diversity of prion strains/types. Analysis of atypical/Nor98 scrapie in the tg338 mouse line is a good example of this. This mouse line over-expresses ovine VRQ PrP (containing amino acids VRQ at positions 136, 156 and 171 respectively) and whilst conventional inbred wild type mice are refractory to infection by atypical/Nor98 scrapie isolates, tg338 mice are highly sensitive and replicate the PrP^Sc^ molecular phenotype that is seen in small ruminants with atypical/Nor98 scrapie [47]. In addition, Thackray and co-workers detail the use of transgenic mouse lines tg338 and tg59, which express either VRQ or ARQ ovine prion respectively, in characterising scrapie isolates that do not transmit to wild type mice [48]. Using these two lines they were able to isolate unique scrapie strains at secondary passage.

Mice transgenic for bovine PrP^C^ have been used to demonstrate the specific features of L-BSE, H-BSE and classical BSE (C-BSE). Masujin and co-workers [49] describe the inoculation of bovinised mice with either L- or C-BSE. Whilst wild type mice were refractory to infection with L-BSE, the transgenic mice developed clinical disease to both L- and C-BSE, with the retention of the same biochemical features that distinguish these two prion types in cattle brain. Similarly, H-BSE has been used to successfully infect bovinised mice and retained its characteristic molecular phenotype [50]. Together, data demonstrate that the three BSE types can be differentiated in bovinised mice [48,49,51], providing a method to type BSE material from suspect atypical bovine cases. 

Transmission of CWD prions to wild type mice lines is inefficient. However, mice expressing cervid PrP^C^ [52] have high attack rates and low incubation times with cervid derived CWD material allowing the analysis of this disease in a rodent model. 

### 2.3. Bioassay in Other Model Species

Alternate rodent models have been developed for the monitoring for prion infectivity and prion type. In recent years the bank vole has been demonstrated to have a high susceptibility to different prion types from a variety of sources [53,54]. The bank vole model is often more sensitive than murine models and can have very short incubation times. This model has been demonstrated to be useful in cases where a scrapie isolate was not able to infect conventional or transgenic murine lines [54]. The brain lesion scoring profiles could again differentiate between two different scrapie isolates and these profiles were maintained upon sub-passage of the inoculums. Recently the bank vole has been used to isolate a type of CWD exhibiting an extremely low incubation time on secondary passage, with incubation periods down to 25-28 days [53]. In a study by Raymond and co-workers [55], multiple CWD isolates were inoculated into both transgenic mice and also different species of hamster. The study demonstrated that after serial passage in Syrian golden hamsters disease incubation periods of CWD isolates could stabilise to either CWD with a short or long incubation period, which was indicative of two different CWD strains. More recently, data using a ferret bioassay also demonstrated the hallmarks of two different CWD types in material originating from mule deer [56]. Apparent differences included incubation time and PrP^Sc^ deposition within the CNS and periphery.

## 3. Histological and Immunohistochemical Analysis of Tissue from Natural Hosts

### 3.1. Histology

Vacuolation patterns are routinely used in mouse bioassay (often following repeat sub-passage) as a diagnostic feature of a prion strain/type. Different prion types can influence lesion profiles in sheep scrapie as well, for example when compared to natural classical scrapie, SSBP1 experimental scrapie produces relatively few lesions in the medulla oblongata [57,58]. However, vacuolation patterns seen in classical sheep scrapie are variable and are influenced by not just prion type but also host *PRNP* genotype [58]. Atypical/Nor98 scrapie produced vacuolation and PrP^Sc^ distribution patterns that were distinct from those seen with classical scrapie: vacuolation is primarily in the cortex of the cerebellum and cerebrum and not the medulla oblongata [33,59]. Amyloidosis is relatively rare in sheep with scrapie [60] and is even more rare in cattle BSE [61]. Intraneuronal vacuolation is often observed in cattle BSE but this is not restricted to BSE infections [62]. The lesion profile is consistent for natural infections of cattle with classical BSE indicating a single prion type is involved in these infections [63,64]. However, there are higher levels of vacuolation and PrP^Sc^ accumulation in caudal brainstem in clinically affected compared to asymptomatic BSE-infected cattle [65].

In goats, BSE and CH1641 (an experimental type of scrapie with a PrP^Sc^ molecular phenotype very similar to BSE PrP^Sc^; [66]) produce similar vacuolation patterns to the classical scrapie SSBP1 [57,67]. 

For CWD infections of deer or elk, again lesions are routinely found in various brain regions although within elk the lesions are generally less severe. In deer, pathology is usually found in the medulla oblongata, olfactory bulb, cortex and hypothalamus (reviewed in [63]). 

Overall, whilst vacuolation patterns are a diagnostic feature of a prion strain/type in rodent bioassays, they have very limited application in the definition of prion types in natural infections due to high variability [58,68,69].

### 3.2. Immunohistochemistry

With BSE in cattle the accumulation of PrP^Sc^ is largely consistent between individuals when analysing distinct neuroanatomical sites and distinct patterns are observed for different BSE types [63,64]. L-BSE (or BASE) produces characteristic amyloid plaques as well as a distinct distribution of PrP^Sc^ compared to C-BSE: L-BSE PrP^Sc^ is present at comparatively lower levels in brainstem and higher levels within more rostral sites of the brain such as the olfactory bulb and olfactory cortex as well as the hippocampus [29]. At present it is not possible to distinguish C-BSE and H-BSE based on PrP^Sc^ deposition [65]. 

Considerable research effort has gone into using IHC to distinguish experimental BSE infections in sheep from other scrapie types. There is no one anatomical location/PrP^Sc^ type that can diagnose BSE from classical scrapie [70]; however, the defining of PrP^Sc^ morphological types, cell-type associations as well as levels of PrP^Sc^ accumulation across multiple neuroanatomical sites allows the discrimination of ovine BSE, CH1641, atypical/Nor98 scrapie and classical scrapie. This technique has been termed PrP^Sc^ profiling and has been pioneered by Jeffrey and co-workers [66,68,71]. Seven distinct morphological types of PrP^Sc^ accumulation have been described, each associated with distinct cell types: four associated with cell surface or extracellular PrP^Sc^ and three with intracellular PrP^Sc^ (reviewed in [68]). Ovine BSE infections could be distinguished from CH1641 and classical scrapie infections with BSE producing relatively high levels of intraneuronal, intramicroglial and neuropil-associated PrP^Sc^ [68,72]. The distinctive BSE profile was not affected by the sheep breed, *PRNP* genotype or route of inoculation of the agent; however, the latter two parameters affected the overall levels of PrP^Sc^ accumulation [72]. Certain classical scrapie isolates/experimental types can also be differentiated via PrP^Sc^ profiling due to distinct cell tropisms and PrP processing [71]. However, scrapie type and host *PRNP* genotype affect the PrP^Sc^ profile and the extent of PrP^Sc^ accumulation [68,71,73].

The analysis of both intra- and extracellular PrP^Sc^ with a range of antibodies to defined epitopes across the length of PrP (PrP^Sc^ epitope mapping) is a distinct technique that also displays a high level of discrimination [32]. Using this epitope mapping technique, clinical ovine BSE cases and some preclinical cases can be distinguished from scrapie by lower levels of intracellular PrP^Sc^ containing the 84-102 amino acid region in dark zone tingible body macrophages (TBMs) of the LRS [74,75] and in neurones and glia in the brain [75]. In addition, intraneuronal levels of PrP^Sc^ were consistently higher for ovine BSE than scrapie across multiple brain regions when detected with a range of antibodies [74]. Interestingly, the exact N-terminal cleavage site for PrP^Sc^ for ovine BSE appeared to be determined by the cell type with three distinct sites being identified, all of which result in smaller PrP fragments compared to classical scrapie PrP^Sc^ [75]. The scrapie type CH1641 displays a ‘peptide map' for intraglial and intraneuronal PrP^Sc^ that is distinct from both classical scrapie and ovine BSE as it is truncated even further from the N-terminus than BSE [66]. However, an independent study indicated that experimental CH1641 and CH1641-like field isolates may well differ [76]. 

With atypical/Nor98 scrapie the pattern of PrP^Sc^ accumulation was distinct from classical scrapie with increased accumulation in the cerebellar cortex and no accumulation in the medulla oblongata [59]. Such cerebellar PrP^Sc^ staining was of a diffuse granular type that has not been seen with classical scrapie [59]. In addition, for atypical/Nor98 scrapie there was no accumulation of PrP^Sc^ in the LRS [33]. 

With goat scrapie, very little PrP^Sc^ staining was seen with SSBP1 or CH1641 infections, whereas goats infected with BSE show clear PrP^Sc^ accumulation particularly in the thalamic, hypothalamic nuclei and in the basal ganglia [57]. Almost identical peptide mapping was observed for intracellular PrP^Sc^ when comparing goat and sheep infections, with intraneuronal PrP^Sc^ for BSE being truncated further from the N-terminus compared to classical scrapie [32]. Interestingly, this peptide mapping technique revealed a natural case of goat BSE [32]. 

### 3.3. Diagnostic Applications of Histology and Immunohistochemistry

For classical BSE, classical scrapie and CWD the presence of spongiform lesions and PrP^Sc^ accumulation in the medulla oblongata at the level of the obex make this region important for the diagnoses of prion disease. The caveat to this is that this region is less involved in pathology in atypical/Nor98 diseases and therefore other brain regions require examination, such as the cerebellum in sheep [35]. 

Whilst vacuolation profiles are consistent for BSE infections in ruminants, other prion diseases of these species produce highly variable profiles indicating the very limited application of this technique for prion type identification. However, this technique may have an application in distinguishing atypical/Nor98 and classical scrapie infections. In contrast, IHC analysis shows great promise in defining prion types in the natural host. L-BSE and C-BSE can be distinguished by IHC examination as can atypical/Nor98 and classical scrapie infections. The PrP^Sc^ profiling and PrP^Sc^ epitope mapping techniques provide highly discriminatory descriptions of PrP^Sc^ phenotypes capable of differentiating BSE, CH1641 and classical scrapie infections in small ruminants. It has also been suggested that these techniques may even be able to define distinct classical scrapie types in the natural host [69,77]. Potential drawbacks to these techniques are that they are relatively laborious, include subjective interpretation of PrP^Sc^ staining patterns and require the availability of high quality tissue covering an extensive range of neuroanatomical sites. For PrP^Sc^ profiling, some of these issues have been addressed by the simplification of the technique by analysing more restricted neuroanatomical areas (within the telencephalon) [69,77].

## 4. Protease Fragmentation of PrP^Sc^ and Glycoform Ratios

One of the main properties that can differentiate PrP^Sc^ from PrP^C^ is the high proteolytic resistance of PrP^Sc^. Of course, this trait is not absolute and it is likely that for all prion diseases PrP^Sc^ will exist as both protease-resistant (PrP^Sc^-res) and protease-sensitive (PrP^Sc^-sen) conformers. At one extreme are 263K scrapie infection in hamsters and human Gerstmann–Sträussler–Scheinker syndrome P102L infection of transgenic 101LL mice, where both infection models accumulate high infectivity titres in the absence of PrP^Sc^-res [78]. However, in all other cases, prion diseases produce levels of PrP^Sc^-res that are detectable by conventional immunoassays. As such, immunoassay detection of PrP^Sc^-res has become the mainstay of prion diagnostics including defining prion types in ruminant diseases. When detected in western blots, the pattern of the PrP^Sc^-res fragments can be used to differentiate prion types as it is thought that their distinct PrP^Sc^ conformers will present distinct cleavage sites to the protease (Figure 1). 

### 4.1. Differentiating Prion Isolates in Small Ruminants

By far the most common protease used in prion typing is PK, which digests PrP^C^ fully but only digests the N-terminal region of PrP^Sc^-res resulting in un-, mono- and di-glycosylated protease resistant core. Hill and co-workers reported [79] that BSE in cattle, when passaged in sheep, displayed a lower molecular weight unglycosylated PrP^Sc^ band compared to classical scrapie. One aspect that needs to be taken into account when applying this method is that the metal occupancy of the PrP^Sc^ [80] and the pH of the sample [81] can influence the apparent molecular weight of the banding pattern. It has also been noted that CH1641 scrapie produces a banding pattern indistinguishable from ovine and caprine BSE [79,82,83]. This method was further developed by Stack and co-workers [84] who used the antibody P4 to detect cleavage products. This antibody binds in the region that is largely removed by PK cleavage of ovine BSE PrP^Sc^ (and CH1641 PrP^Sc^) but retained during scrapie PrP^Sc^ digestion. When compared to an antibody binding further C-terminally in the protease resistant core, ratios of P4: “core antibody” are much higher for classical scrapie that for BSE or CH1641. 

It was also noted that on western blots the glycoform pattern of the PrP^Sc^-res triplet bands could be indicative of prion type, for instance with bovine and ovine BSE displaying a dominant diglycan compared to classical scrapie [79]. The robustness of using glycoform patterns for defining prion type has been the subject of much debate with some studies reporting that for certain rodent passaged prion strains, the glycoform profile of PrP^Sc^ can be influenced by host factors such as the brain region and *PRNP* genotype [85,86]. However, other studies have found consistent glycoform profiles across different brain regions [83,87,88] and in sheep with distinct *PRNP* genotypes [87]. Researchers have suggested that due to high variation between blots, glycoform profiles need to be derived from repeated analysis of samples across multiple blots using photo-imaging techniques to quantify band intensities [83,88]. It has also been noted that the glycoform profile will be influenced by the antibody used to detect the prion protein, which needs to be taken into account when comparing data between studies [83,89]. Whilst glycoform profiling is a useful diagnostic tool, it is difficult to perform and does not always show distinction between classical scrapie and BSE, or between CWD and scrapie [90]. 

Screening of small ruminant prion diseases has mainly been designed to distinguish the zoonotic agent BSE from the non-zoonotic agents of scrapie. Most studies use a combination of molecular weight of PK-resistant PrP^Sc^, the ratio of P4:‘core antibody’ binding and glycoform profiling to search for BSE infections. Samples displaying one or more traits of BSE are usually subsequently analysed by mouse bioassay for confirmatory prion typing [84,89,91]. Given the experimental designs, it is still largely unknown what molecular variations exist for classical scrapie in the natural hosts [82,83,91]. 

In addition to PK cleavage, an alternative protease thermolysin can also be applied to distinguish BSE from classical scrapie in small ruminants. Digestion resulted in distinct cleavage patterns of PrP^Sc^ for ovine BSE compared to classical scrapie that was not influenced by host genotype or scrapie type [92].

Recently, there have been further developments in the differentiation of BSE from natural scrapie with particular emphasis on differentiating BSE from CH1641 which is not possible using any of the criteria described above [84]. Pirisinu and co-workers [93] demonstrated that ovine BSE was more stable to guanidinium hydrochloride (GndHCl) than classical scrapie or CH1641 scrapie and that treatment with 3.5 mol/L GndHCl before digestion in combination with P4/’core antibody’ binding ratios allowed the discrimination of both CH1641 and classical scrapie from ovine BSE. Alternatively, the probing of a single western blot with differently labelled SAF84 and L42 antibodies revealed a so-called dual glycotype aspect at the size of the monoglycosylated band which distinguished CH1641 from BSE in sheep; as CH1641 had a relatively high SAF84 to L42 binding ratio compared to BSE [8]. In addition, a microwell immunofluorometric assay has been developed to differentiate between classical scrapie, atypical scrapie, CH1641 scrapie, experimentally infected ovine BSE and naturally infected bovine BSE [94]. The assay is based on the capture of PrP^Sc^-res with three distinct antibodies that bind to different regions on PrP. Due to distinct PK-cleavage sites for PrP^Sc^ from different TSE types, all three antibodies capture PrP^Sc^-res from classical scrapie, two bind PrP^Sc^-res from atypical scrapie and a different combination of two antibodies bind to PrP^Sc^-res from BSE and CH1641. BSE and CH1641 can then be differentiated due to the distinct binding ratios of these two antibodies.

Endogenous proteolysis also results in PrP^Sc^ fragments that may also be indicative of prion type in ruminant, such as the so-called C2 fragment in scrapie cases but not in experimental BSE or CH1641 cases [92] and the diagnostic 14kDa fragment (so called CTF14) that is detected with the antibody SAF84 in experimental CH1641 and naturally occurring CH1641-like isolates [95].

Atypical/Nor98 scrapie cases were first noted by Bernestad and co-workers [33], this can occur in both sheep and goats [36]. Atypical/Nor98 scrapie produces PrP^Sc^-res with a lower protease resistance than classical scrapie and PK digestion patterns for atypical/Nor98 scrapie often (but not always, [34]) also contain diagnostic fragments between 6.5 and 12 kDa [96].

### 4.2. Differentiating Cattle BSE Types

The analysis of PK cleavage patterns of PrP^Sc^ has led to the description of variants of BSE in cattle. Biacabe and co-workers [30] reported higher molecular weight fragments compared to classical BSE, and named this atypical type H-BSE. This was shown to be due to cleavage of the atypical PrP^Sc^ further N-terminally than in classical BSE, resulting in the efficient retention of the 12B2 epitope and therefore relatively high levels of binding to this antibody [97]. Casalone and co-workers [29] reported a BSE type producing a lower molecular weight PrP^Sc^-res, termed BASE or L-BSE. In addition to the differences in PK cleavage sites, L-BSE can be distinguished from C-BSE and H-BSE by a distinct glycoform pattern [27,28] although as mentioned previously, glycoform profiling of prion types can be difficult to perform reproducibly [83,85,86,88]. Also, the H-BSE appears to contain two distinct glycoform profiles depending on the antibody used to detect PrP [27] that can be exploited to diagnose this type when using the so called dual glycotype aspect for detection [8]. H-BSE also produces a unique fragment of 6-10kDa [98]. Whilst the molecular weight and antibody recognition of H-BSE PrP^Sc^-res is sufficient to diagnose such infections, the difference in molecular weight between L-BSE and C-BSE PrP^Sc^-res is just 0.3kDa for the unglycosylated band and is not suitable for routine prion typing [27]. However, Jacobs and co-workers [27] demonstrated that both H-BSE and L- BSE display enhanced protease digestion at pH8 relative to pH6.5 compared to C-BSE which may provide a more robust biochemical test for distinguishing L-BSE and C-BSE types.

## 5. Differential Stability of Prion Types

The underlying theory that prion characteristics are defined by the conformational make-up of the PrP^Sc^ molecules led to the development of tests to characterise the stability of these conformers. These methods were developed into two complementary assays that quantified the disruption of prion conformations with GndHCl [12,99]. The conformation-dependent immunoassay (CDI) analysed the exposure of epitopes within PrP^Sc^ molecules upon denaturation and the conformational stability assay (CSA) measured the loss of PK-resistance of the PrP^Sc^ molecules upon denaturation. 

The CDI was developed and applied to rodent passaged strains and could distinguish multiple prion strains in hamsters [12]. Despite this obvious promise, the application of the assay for typing of ruminant prions has not been pursued.

**Figure 3:**
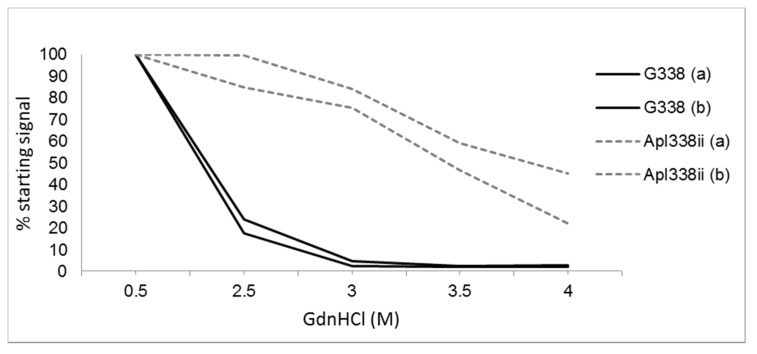
Conformational stability assay of two scrapie strains (as determined in Tg338 mice). Two isolates of each strain G338 and Apl338ii were compared by western blotting after treatment with increasing concentrations of the denaturant Guanidine hydrochloride (GdnHCl). Denatured samples were treated with proteinase K and then western blotted using a core antibody. Total PrP^Sc^ signal was calculated by densitometry and plotted as a % of the PrP^Sc^-res signal after treatment with 0.5M GdnHCl. These two scrapie strains have very different sensitivities to GdnHCl, G338 being less stable than apl338.

The CSA measures the stability of PrP^Sc^-res (Figure 3) and a particular prion type can be defined as the [GndHCl]_1/2_ value: the concentration of GndHCl that results in the denaturation of half of the PrP^Sc^. The determination of such values could differentiate some but not all hamster passaged prion strains [100]. With respect to ruminant prions, CSA analysis has shown that cattle BSE was less stable than a single scrapie isolate but conversely mouse-passaged BSE prion was more stable than passaged scrapie [12]. A further study demonstrated that BSE passaged in sheep was more stable than classical and CH1641 ovine scrapie [93]. It therefore seems that stability is dictated by both the prion type and host species. 

Pirisinu and co-workers [99] also adapted the CSA assay to analyse the detergent solubility of PrP^Sc^ upon treatment with a range of GndHCl concentrations, a process termed the conformational solubility and stability assay (CSSA). With this CSSA assay, different ovine classical scrapie isolates with a range of *PRNP* genotypes could be differentiated from a similarly diverse set of atypical/Nor98 scrapie isolates.

Overall, the analysis of the stability of prion conformers can be monitored through changes in epitope accessibility or protease-resistance, and total PrP can be analysed directly or following separation of PrP^Sc^ from PrP^C^. Such analyses show significant promise in defining prion types and correlating structural properties of the prion with disease pathologies such as the duration of asymptomatic or clinical phases of disease. However, relatively little data is available to date on the application of such assays to ruminant samples.

## 6. Cell Culture Methods

A number of cell lines permissive to prion multiplication have been developed including established cell lines, neuronal stem cells, and primary neuronal cells. Whilst initial cell lines were only permissive to rodent-adapted prion strains, the rabbit epithelial RK13 cell line expressing an ovine PrP^C^ gene was permissive to ovine scrapie and was found to accumulate high levels of PrP^Sc^ [101]. Since this study, prion replication has been demonstrated in a variety of cell systems. An isolate from CWD has been successfully transmitted to a mule deer cell line [102], and cell lines expressing ovine PrP (Rov9 and MovS6) have been infected with experimental and natural ovine scrapie agents [103]. Cell models of prion infection are comprehensively reviewed by Vilette [104].

The standard scrapie cell assay (SSCA) involves the use of susceptible N2a cells which are exposed to prion containing samples for 3 days, grown to confluence, split 1:10 three times, then the proportion of PrP^Sc^ containing cells determined by an ELISA using ELISPOT plates with automated counting. [105]. The SSCA has also been combined with the use of the Rov9 and MovS6 cell lines, which both express the ovine PrP VRQ allele [103]. The Rov9 cell line could be used to estimate the infectious titre of an ovine scrapie brain pool. The SSCA has been further adapted for use in the discrimination of rodent-passaged scrapie strains based on the findings that some cell lines show preferences for certain strains [105]. However, given that there are only a very limited number of cell lines permissive to ruminant prions and even then they are only infected by a limited number of isolates, it seems unlikely that this prion typing platform will be applicable to ruminant isolates in the near future.

## 7. *In Vitro* Replication of Prion Types

**Figure 4:**
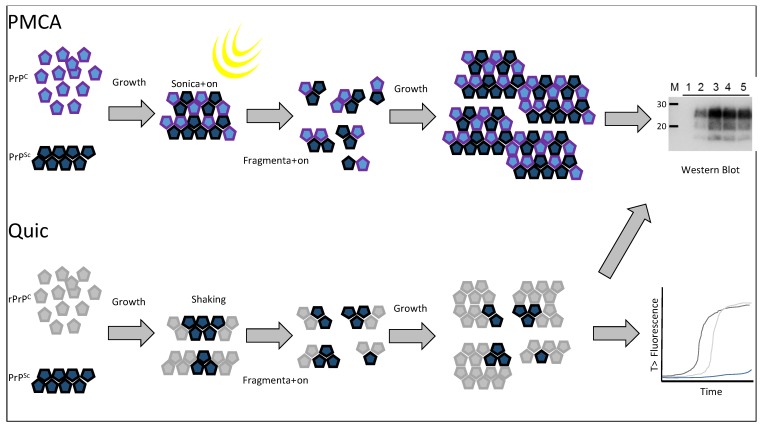
Protein Misfolding Cyclic Amplification (PMCA) is an *in vitro* reaction thought to reproduce the molecular events of the PrP^C^ to PrP^Sc^ conversion that occur *in vivo* with transmissible spongiform encephalopathies (TSE) diseases. Here a PrPC substrate (a healthy brain homogenate) is seeded with a PrP^Sc^ source, which causes conversion of PrP^C^ to further aggregates of PrP^Sc^. These aggregates are fragmented into a number of smaller seeds by sonication and the process of prion growth is reinitiated. Over time enough PrP^Sc^ can be produced to be detected by western blot. Quaking induced conversion (Quic) is an alternative *in vitro* assay that is also based on the seeding ability of PrP^Sc^. A PrP^Sc^ seed is used to convert a recombinant PrP^C^ substrate into an “amyloid” form. These large aggregates are fragmented into smaller seeds by vigorous shaking followed by further re-seeding and growth. Quic reactions are generally monitored by Thioflavin T fluorescence over time but the products can also be measured by western blot.

A non-radioactive cell free conversion assay that used recombinant mouse PrP as the substrate to examine the replication of murine passaged scrapie strains and BSE demonstrated that the conversion efficiency differed between some strains [106]. More recently, the protein misfolding cyclic amplification (PMCA) methodology has demonstrated distinct replication traits for prion types across multiple studies. PMCA is an *in vitro* prion replication assay that relies on iterative rounds of sonication and incubation [107] (Figure 4). The sonication step is thought to break prion aggregates into smaller units that can efficiently seed the conversion of PrP^C^ into PrP^Sc^ during the incubation period. The technique allows the amplification of minute amounts of PrP^Sc^ when spiked into normal brain homogenate substrate where the prion is amplified over multiple (24–48) cycles of sonication/incubation (a round) and the substrate is then replenished by diluting the amplification products into fresh substrate. Any number of rounds can then be carried out. When analysing a range of rodent passaged scrapie strains or synthetic prions, Gonzales-Montalban and co-workers [108] demonstrated that the addition of beads to the PMCA reaction (PMCAb) had distinct effects on replication efficiency that was strain dependent. Strains with higher conformational stability displayed more enhancement of replication efficiency with the addition of beads compared to strains with lower conformational stability. Further analyses demonstrated that the prion strain influenced both the growth rate of PrP^Sc^ particles and also the total yield of PrP^Sc^ produced in the assay [109]. Other studies using rodent passaged prion strains have shown that changes in the substrate/cofactor availability during the PMCA/PMCAb reactions can also influence the amplification efficiency in a strain-specific manner. Such changes have included the use of RNase treated substrate or supplementation of substrate with polyA, polyG or DNA [110], as well as the glycosylation state of the PrP^C^ substrate [111]. 

In terms of the analysis of prions from their ruminant hosts, PMCA has been applied to a range of ovine scrapie types as well as ovine passaged BSE. Taema and co-workers [112] demonstrated that ovine BSE was amplified within 5 PMCA rounds using either VRQ/VRQ or AHQ/AHQ substrate and CH1641 scrapie did not amplify in either substrate. In addition, the use of AHQ/AHQ substrate did not support the amplification of any classical or atypical/Nor98 scrapie isolates from sheep with a range of *PRNP* genotypes. These observations have been developed into a sensitive assay using a combination of the VRQ/VRQ and AHQ/AHQ brain homogenate substrates [113]. These two substrates encourage the amplification of BSE over scrapie and when used in sequential rounds of PMCA have demonstrated amplification specificity to BSE, even when BSE is present with a vast excess of different scrapie isolates [113,114]. The assay was shown to be far superior to ELISA and western blot tests for detecting ovine BSE in the presence of a large excess of scrapie agent [114]. Such an assay could be applied to monitor the status of animals for mixed infection where the levels of BSE may be very low, and whose detection may be obscured by the presence of scrapie. The ability of PMCA to distinguish ovine prion types is further supported by data showing that classical scrapie isolates from ARQ/ARQ sheep produced two distinct amplification patterns when amplified in ARQ/ARQ and VRQ/VRQ substrates [115]. These amplification patterns were almost always consistent for flock-mates indicating the presence of distinct prion types. In addition, classical scrapie type MRI could be distinguished from classical scrapie types SSBP1 and Dawson by amplification patterns in these two substrates. Overall, PMCA has shown application in distinguishing ovine prion types by analysing amplification efficacy in PrP^C^ substrate with distinct *PRNP* genotypes. 

Together, the data supports the hypothesis that a range of substrate/co-factor conditions in tandem with a range of amplification conditions could provide a panel of defined PMCA conditions capable of defining prion types in the natural host; effectively producing an “amplification profile” for each ruminant prion type. 

Recently, the use of recombinant bank vole PrP as a universal substrate for a similar *in vitro* replication assay known as quaking induced conversion or “Quic” has been described [116] (Figure 4). Quic differs from PMCA in that fibrils of recombinant prion are generated during cycles of incubation followed by shaking which is thought to break growing fibrils and form the new nucleation sites for fibril formation. The products of the reaction are formed from the seeding effect of prion containing material; however unlike PMCA the products of this reaction are non-infectious. The authors demonstrate some specific differences in the PK digestion profiles on western blots of Quic reaction products that were seeded by different ruminant prion types, demonstrating that *in vitro* amplification and prion typing of unknown samples may be possible using this rapid and sensitive technique.

## 8. Conclusions and Future Perspectives

Whilst the cases of BSE in ruminants is now very low and the associated concern for the contamination of the human food chain with the zoonotic BSE agent has eased, there are still concerns surrounding the exposure of humans to prions from food-producing animals. The more recent description of atypical/Nor98 scrapie in goats/sheep and atypical bovine BSE as well as the discovery of two distinct types of CWD all raise the possibility that further types of prions are circulating in ruminants that are not detected and/or defined by current assay methods. An additional concern is that novel types may emerge in these animals. One diagnostic challenge in prion biology is to develop and apply prion typing tests to fully elucidate the range of existing prion types in ruminants and to monitor for the emergence of novel types. This is a significant challenge as it is unknown what molecular and pathological differences any novel type will have compared to those already described. Therefore, assays that have a wide range of distinct measurements that describe a PrP^Sc^ type or *in vivo* pathology will be best suited for diagnosing new prion types. 

There have been significant advances in recent years in *in vitro* prion typing assays. The development of PrP^Sc^ profiling and PrP^Sc^ epitope mapping provide IHC methods that can differentiate classical, atypical/Nor98 and CH1641 scrapie from BSE in sheep and has the potential to further discriminate between classical scrapie types. The methods examine multiple anatomically distinct brain regions and cellular prion locations and/or map multiple epitopes of PrP^Sc^. The methods therefore have a wide range of possible test outcomes that are examined and have great diagnostic application. The drawbacks of these methods are that they are labour intensive and require extensive good quality brain material. Prion typing tests are almost certainly going to be most applicable to post-mortem derived tissues from routine sampling sites in animals confirmed as having a prion disease. Therefore, the application of these IHC methods using routine surveillance samples to test for novel or existing types is perhaps limited. The examination of proteolytic fragments of PrP^Sc^ by western blotting has formed the mainstay of high throughput prion typing assays for ruminants. Recent advances using multiple antibody probes for single western blots along with the partial denaturation of samples prior to proteolysis have improved the diagnostic capability of such tests. However, these assays have each been specifically designed to differentiate defined existing prion types by looking for a specific fragmentation pattern and therefore may be limited in their application to detecting novel prion types. In contrast, the CSA measures the stability of a prion type over a range of denaturation conditions and therefore may well define novel types with distinct stabilities. It could be envisaged that the use of multiple denaturation conditions within CSA combined with epitope mapping of the resulting PrP^Sc^ fragments by western blot could provide a range of test outputs that measure the conformation of PrP^Sc^ to define types. A limitation of PrP^Sc^ analysis in all of these methods may be that PrP^Sc^ can display variation in its neuroanatomical location dictated by the prion type pathology and sufficient PrP^Sc^ may not be present at the routine sampling sites to allow analysis by these methods. A methodology that could detect prion types at very low concentration would therefore be advantageous. PMCA may provide such a method. It has been reported that PMCA usually maintains the PrP^Sc^ molecular profile of the seed and is exquisitely sensitive in detecting PrP^Sc^. A combination of PMCA amplification followed by analysis of the PrP^Sc^ product by an assay such as CSA may allow a robust prion typing assay to detect novel types even when PrP^Sc^ is present at low levels in the tissue sample. Alternatively, PMCA methods have now also been directly applied to prion typing in ruminants demonstrating that different prion types display different replication efficiencies under defined conditions. The data supports the hypothesis that a range of substrate/co-factor conditions in tandem with a range of amplification conditions could provide a panel of defined PMCA conditions capable of defining prion types from the natural host; effectively producing an ‘amplification profile’ for each type. 

For all *in vitro* prion typing assays the definition of a novel prion type would need to be assessed as being representative of an inheritable *in vivo* strain phenotype rather than of a quasispecies that was selected/identified during the *in vitro* analyses. Therefore, any novel type would require analysis within rodent bioassay to define a novel and consistent pathology that is stable upon sub-passage and can therefore be described as a novel prion strain. The recent advent of transgenic murine models and a range of rodent host species have extended the use of rodent bioassay to a relatively wide range of natural ruminant isolates making this a much more realistic possibility.

## Abbreviations

Prion protein: PrP; disease associated prion protein: PrP^Sc^; cellular prion protein: PrP^C^; variant Creutzfeldt Jakob disease: vCJD; bovine spongiform encephalopathy: BSE; chronic wasting disease: CWD; transmissible spongiform encephalopathy: TSE; central nervous system: CNS; lymphoreticular system: LRS; proteinase K: PK; immunohistochemistry: IHC; bovine amyloidotic spongiform encephalopathy: BASE; guanidinium hydrochloride: GndHCl; conformation-dependent immunoassay: CDI; conformational stability assay: CSA; the concentration of GndHCl that results in the denaturation of half of the PrP^Sc^: [GndHCl]_1/2_ value; conformational solubility and stability assay: CSSA; Protein Misfolding Cyclic Amplification: PMCA; PMCA including beads: PMCAb.
